# Supplementary material for: Developing an organizational capacity assessment tool and capacity-building package for the National Center for Prevention and Control of Noncommunicable Diseases in Iran
Source: PLoS One. 2023 Jun 29;18(6):e0287743. doi: 10.1371/journal.pone.0287743 (PMC10309984; doi:10.1371/journal.pone.0287743)
Supplement: S1 Appendix — (DOCX) [file pone.0287743.s001.docx]

**Box A. Reviewed documents**

| Reviewed documents | WHO-PEN, best buys, and other recommended interventions. IraPEN, National Action Plan for Prevention and Control of Noncommunicable Diseases and the Related Risk Factors in the Islamic Republic of Iran, 2015-2025, National Physical Activity Plan for Health Promotion in IR Iran, National Diabetes Prevention and Control Program, Comprehensive Tobacco Control Act, National Nutrition and Food Security Document 2012-2020, Comprehensive program for the prevention, treatment and reduction of alcohol-related toxicity, National policies related to sugar, salt, and fat, National guidelines for COPD, prevention, diagnosis and treatment guidelines, National policies related to physical activity, nutrition, tobacco, and alcohol, clinical services. |
| --- | --- |

**Table 1. The individuals with expertise who worked on the tool design phase**

| Executive or scientific experience | Number |
| --- | --- |
| Manager of NCDs and related risk factors at the national level | **8** |
| Director of research and development )MoHME) | **4** |
| The professionals in the fields of human resources, finance, administration, and communication | **8** |
| Total | **20** |

**Table 2. The expert team number two for the tool's validity and reliability**

|  | Executive or scientific experience | Number |
| --- | --- | --- |
| Validity | Administrator of the organization at the national and provincial levels (health sector) | 3 (National), 5 (provincial) |
|  | Faculty members from colleges and universities working in the field of health system organizational management | 2 |
|  | Organizational and hospital assessor | 2 |
| Reliability | National manager (Former) and provincial manager for Physical activity | 1+1 |
|  | National manager (Former) and provincial manager for Cardiovascular disease | 1+1 |
|  | National manager (Former) and provincial manager for Tobacco | 1+1 |
|  | Total | 12+6=18 |

**Table 3. Characteristics of interviewees (Expert Team three) for qualitative phase**

| Executive or scientific experience | 1 | 2 | 3 | 4 | 5 | 6 | 7 | 8 | 9 | 10 | 11 | 12 | 13 | 14 | 15 | 16 | 17 | 18 | 19 | 20 | 21 | 22 | 23 |
| --- | --- | --- | --- | --- | --- | --- | --- | --- | --- | --- | --- | --- | --- | --- | --- | --- | --- | --- | --- | --- | --- | --- | --- |
| National or provincial policy maker/manager |  |  |  |  | * | * |  |  | * | * | * | * | * | * | * | * | * | * | * | * | * | * | * |
| Academic | * | * | * | * | * |  |  |  |  |  |  |  |  |  |  |  |  |  |  |  |  |  |  |
| Healthcare provider |  |  |  | * |  |  |  | * |  |  |  |  |  |  |  |  |  |  |  |  |  |  |  |
| Civil Society |  |  |  |  |  |  | * | * |  |  |  |  |  |  |  |  |  |  |  |  |  |  |  |

- **Designing Tool (Organizational Capacity Assessment Tool)**

**2.1 Reviewing relevant literature:**

Based on relevant literature on capacity assessment and capacity building, the tools have differences in construction, questions, assessment description types, capacity domains, and scoring options. We noticed the following tools (Fig 1) are the most frequently referenced and will become our tool's basis.

Box B: Comprehensive review

| In order to answer the following question, a comprehensive literature review was conducted:  #4Objectives: What are the dimensions, processes, challenges in organizational capacity measurement/ capacity building tools and processes?  1. Search strategy and keywords  (("Capacity"[Title] OR "capacity assessment"[Title] OR "capacity building"[Title] OR "performance"[Title] OR "effectiveness"[Title] OR "development"[Title] OR "learning"[Title]) AND ("Organizations"[Title] OR "Organizational"[Title] OR "Organization"[Title] OR "Organisation"[Title])) AND ((english[Filter]) AND (2000:2020[pdat]))  Furthermore, references from chosen studies were examined. One of the studies examined nearly 300 capacity assessment tool and questionnaires.  DATABASE: PubMed, Scopus, and Web of Science, google scholar, gray literature and website  Eligibility criteria  INCLUSION CRITERIA   - Studies conducted in the developed and developing countries. - Studies that used the capacity assessment for organizations. - Studies published in English. - Studies published in journals with the peer-review system. - Studies published between 2000 up to January 2020. - Articles whose full text was available. - Tools, guidline, and handbook - Tools and studies in the field of health or disease - Studies by prominent institutions including WHO, UN and OECD   EXCLUSION CRITERIA   - Studies conducted for no-organization - Studies published in Non-English language. - Theses and chapters of books. - Individual or professional capacity building   EXTRACTION OF DATA  Two researchers extracted data from a selected study independently. If they cannot reach an agreement, another researcher acts as an arbitrator. Dimensions and sub-dimensions used, scoring processes, evaluation, and key points in designing and implementing capacity assessment were extracted.  DATA ANALYSIS  For the analysis of qualitative data, we used the framework approach.  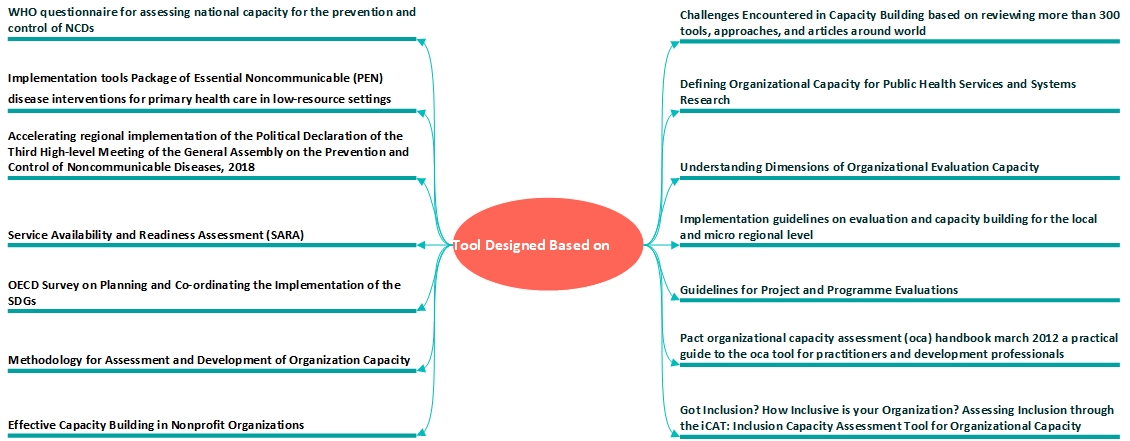Fig 1. The most important literature review cases that have extracted important principles for designing tool [1-14] |
| --- |

**2.2** **Organizational Capacity Assessment Domains, scoring, construction**

Over time, the dimensions of most tools have been fixed between 4 to 12 specific dimensions depending on the organization's type and missions [12, 13, 15].

More than 300 items (websites, tools, articles, reports) related to organizational capacity assessment were reviewed by US Agency for International Development (USAID), and a list of possible domains which have been dealt with in capacity assessment tools was provided [6]. We updated this list with a questionnaire developed by the WHO for assessing organizational capacity in the field of NCDs[14] and other studies (Fig 1), and based on the opinions of the research and expert team; we have divided them into six general domain that makes up the main parts of the tool(Fig 2). Based on the relevant literature and experts' opinions, the subdomains were designed to measure the extent of NCNCD's control over those cases; while the WHO questionnaire measures the presence or absence of factors in most cases, the tool measures NCNCD's authority over the designed subdomains.


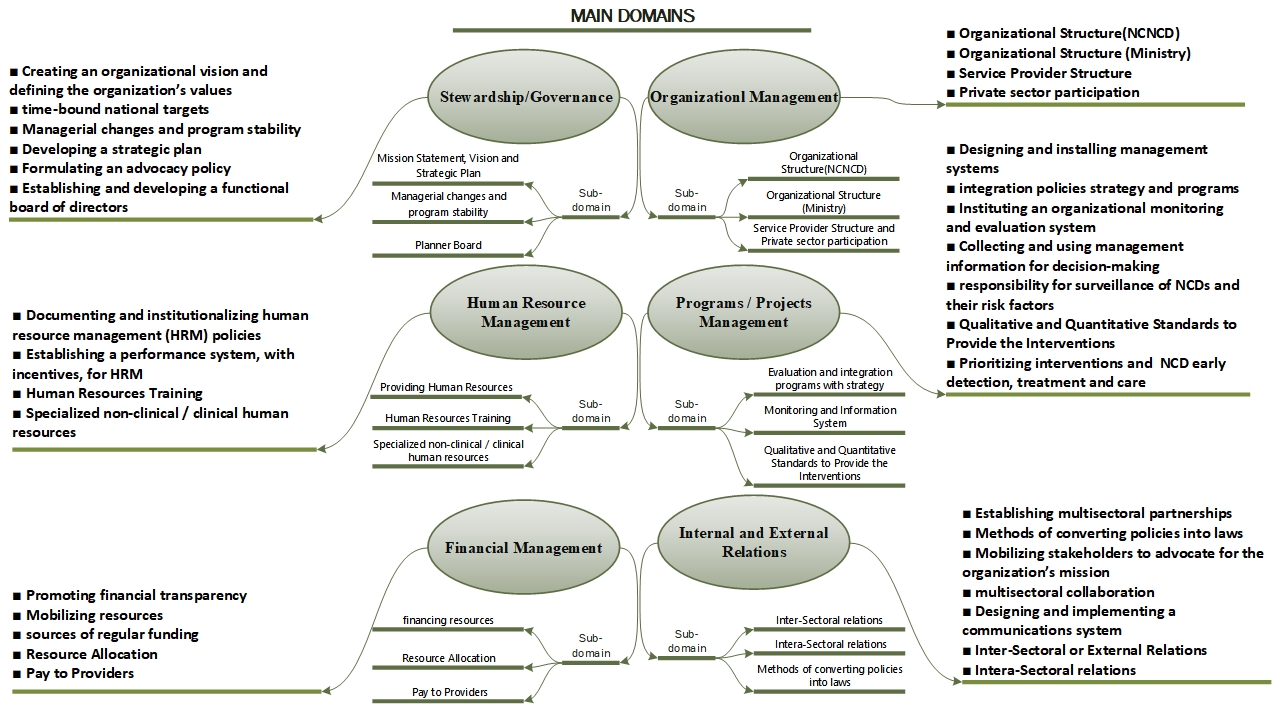


**Fig 2. The six general domain that makes up the main parts of the tool**

**Construction of domains:**

According to Informing Change, a guide to organizational capacity assessment tools [16], the structure of the questions focuses on three sub-domain.

A: Each domain has three questions (sub-domain) for notification see Fig 2,

B: 4 Rubric Response Statements for each subdomain for better understanding,

C: and a column for assessment, which is scored based on Rubric Response see Table 4.

**Table 4 Construction of domains**

| Domain 1/ | Low Capacity | Basic Capacity | Moderate Capacity | Strong Capacity | Score |
| --- | --- | --- | --- | --- | --- |
|  | 1 | 2 | 3 | 4 |  |
| Question 1 | Rubric Response 1 | Rubric Response 2 | Rubric Response 3 | Rubric Response 4 |  |
| Question 2 | Rubric Response 1 | Rubric Response 2 | Rubric Response 3 | Rubric Response 4 |  |
| Question 3 | Rubric Response 1 | Rubric Response 2 | Rubric Response 3 | Rubric Response 4 |  |

**Validity testing:**

Each domain's questions were designed based on reviewing related studies and opinions of the study team and the expert team. The researchers used short, simple sentences and non-specialized words to minimize measurement error. They were reviewed in several rounds to assess the content validity of the questions and statements. The final version of the tool was scored by experts (12 experts with organizational management, human resources management, financial management, public health and organizational relationship management skills). The Item-level Content Validity Index (I-CVI) and The Content Validity Index for Scales (S-CVI) were utilized to calculate the content validity by using the following formulas **I-CVI=** the number of experts giving a rating of either 3 or 4/ the total number of experts; **S-CVI**= the sum of I-CVIs/ the number of items.

If the I-CVI value is higher than 0.79, the item is relevant; if it is between 0.70 and 0.79, it needs modifications; and if it is less than 0.70, the item is removed. Questions were removed, changed, and modified repeatedly until each item for each sub-dimension had acceptable values. Tables 6 to 11 present the values.

CVR: CVR, which assesses an item's essentiality, is the second method of empirical analysis. A higher CVR score indicates stronger experts agreement and ranges from 1 to -1 [17]. The CVR is calculated according to the formula: CVR = (Ne - N/2)/(N/2), where Ne represents the number of experts who marked an item as "essential" and N represents the total number of experts.

The CVR was calculated and, in all cases, was above the minimum value of 0.56 according to Lawshe's content validity ratio [18] and S-CVI Is equal to 0.903 (please see Tables 6 to 11).

Three of the 18 sub-dimensions or tool questions were modified and their validity was assessed again; including subdomains 3.3; 5.1 and 5.3)

**Reliability testing**

For the purposes of reliability, we targeted three deputies of NCNCD, Departments of physical activity, tobacco, and cardiovascular disease. In total, we involved 3 + 6 partners to compare the results of their evaluations with the CohenKappa test. The organization’s results by the NCNCD Center to the tools were compared to the organization’s results by the experts. The experts were well-versed in the target deputy, who were purposely selected. According to a study sponsored by the Rockefeller Institute [19], the general assumption is that if the tool is measuring the results correctly, the expert evaluation score should not be much different from the NCNCD member evaluation results. We are looking for a reliability of 0.4 or higher in this study. Statistical analysis was conducted using R version 3.6.2 [20], package DescTools [21].

Tools for capacity-building have evolved [22], and their measurement, dimensions, validity and reliability [19] have improved over time. Because we used the tool in 7 different sections (1. cardiovascular disease and hypertension; 2. Diabetes;3. Chronic Respiratory Disease; 4. Obesity and Physical Activity; 5.Tobacco and Alcohol; 6. Nutrition (salt, sugar, fat, fruit and vegetable); and 7. cancers) it is not possible to bring seven tools separately in the results section; therefore, We outline the general structure applicable to all seven domains and use numbers instead of some phrases. For example, Instead of the number 1, replace cardiovascular disease and hypertension wherever you see it. Replace numbers with expressions to use the tool.

A list of measures, recommendations, interventions, and programs related to each area(1to 7) was presented to the raters before using the tool to score the sub-domains with these elements in mind. The dimensions and subdomains intended for the tool are shown in Figure 2. For reliability, as Table 5 shows, for 3 aria that we tested, the kappa values show moderate (0.4–0.6) to strong (0.6 and higher) interrater agreement. This means that the tool and its methodology of application meet the reliability standard.. We considered that, if reliable, both raters should indicate the same organizational capacity level. As you can see, this hypothesis has been verified through Kappa statistics.

**Table 5.The reliability of the tool**

| Aria | Rater 1A/1B/1C (from NCNCD) VS. Rater 2A/2B/2C (Experts) | | | Rater 1A/1B/1C (from NCNCD) VS. Rater 3A/3B/3C (Experts) | | |
| --- | --- | --- | --- | --- | --- | --- |
|  | Kappa | Std. Err. | p-value | Kappa | Std. Err | p-value |
| A:Physical activity | 0.7202 | 0.1664 | 0.0000 | 0.5082 | 0.1595 | 0.0007 |
| B: Cardiovascular disease | 0.5337 | 0.1758 | 0.0012 | 0.6289 | 0.1737 | 0.0001 |
| C:Tobacco | 0.6697 | 0.1561 | 0.0000 | 0.5673 | 0.1693 | 0.0004 |

**Cognitive interviews**

The tool was presented to six NCNCD experts, and during cognitive interviews they were asked to describe what was going through their minds while they answered the questions. For each question in the evaluation, three modes were taken into account. 1. The question is not problematic. 2. There is a tiny misunderstanding, and 3. The question is ambiguous. The content of the rubric response has been revised in situations 2 and 3 to avoid any potential misunderstandings. Additionally, the colleague's approval of the grade during the tool grading procedure was taken into account. The respondents had access to the facilitator that was taken into account by NCNCD.

**Domain 1:** **Stewardship/Governance**

Questions to be considered for governance include the following sub-domain: (1.1) Vision, mission statement, and strategic plan; (1.2) Managerial and political changes and avoidance of the effects of the characters (1.3) The main planning and decision-making bodies. [23-36]. The Leadership dimension examines whether there is a formal written strategic plan and whether all matters 1 to 7 have been addressed. It also measures the relevance of programs to the personal opinions of those in power, The dependency of programs on individuals and their lack of evidence-based planning will reduce their stability. It also examines the variety of skills needed in the planning board and their accountability.

Table 6. Sub-domains of stewardship/governance and content validity degree

|  |  | Low Capacity 1 | Basic Capacity 2 | Moderate Capacity 3 | Strong Capacity 4 | Score | **Content Validity** | | |
| --- | --- | --- | --- | --- | --- | --- | --- | --- | --- |
|  |  |  |  |  |  |  | N_e_ / N | CVR | I-CVI |
| 1.1 | The National Center for Noncommunicable Diseases has a mission statement, vision, and written strategic plan related to 1,2,3,4,5,6,7 that are publicly available and guide the MoHME in matters of 1,2,3,4,5,6,7. It is reviewed annually. The Center's strategic plan appropriately addresses the elements and determinants associated with 1,2,3,4,5,6,7. | Currently, the center has no written mission statement, vision, and strategic plan that addressing 1,2,3,4,5,6,7. The center's strategic plan did not refer to 1,2,3,4,5,6,7 -related goals, physical activity, or the strategic plan referred to elements of 1,2,3,4,5,6,7 that are outdated. | The mission statement, vision, and strategic plan of the center are unofficial or unwritten or the mission, vision, and strategic plan of the center has long been written and incompatible with the current goals of the center, particularly those related to 1,2,3,4,5,6,7. They are vague and not known. The center's strategic plan outlines strategies for 1,2,3,4,5,6,7, but they are not clear. | There is currently a clear mission statement and vision and a vision related strategic plan that addresses 1,2,3,4,5,6,7 in writing, which is well-known by center staff and provinces. That guides their actions and plans but is not reviewed annually, and some necessary measures related to 1,2,3,4,5,6,7 have been taken out of the strategic plan. The center's strategic plan refers to strategies related to 1,2,3,4,5,6,7, and the specific strategic plan response to issues related to 1,2,3,4,5,6,7 is clear. But there is a deviation from the strategic program. | There are a clear, updated and well-known mission statement and vision on 1,2,3,4,5,6,7 that is reviewed annually, as well as an updated vision-related strategic plan that guiding the Ministry of Health and Provincial actions in the area of 1,2,3,4,5,6,7 and is reviewed at least annually. The center's strategic plan accurately and clearly states the goals and strategies related to 1,2,3,4,5,6,7, and the specific responses to those goals are clearly identified and there are no deviations from the strategic program. |  | 12/12 | 1.0 | 1.0 |
| 1.2 | To what extent is the strategic plan, (especially 1,2,3,4,5,6,7-related goals) safe from personality domains? For example, considering that the coming of the new government or the Minister of Health will not eliminate the fundamental changes based on the unscientific views of individuals and parties. | Given the lack of stakeholder involvement in strategic planning, it is believed that with the change of policy makers and managers, the strategic plan faces many changes and is not immune from the dominance of personalities. | Given the modest involvement of stakeholders in strategic planning, it is thought that with the change of policy makers and managers, the strategic plan will not undergo structural change, but we will see changes anyway. | Given a large number of stakeholders involved in the preparation of the strategic plan and periodic surveys of obesity-related stakeholders during the executive years of the plan is thought to be changing policymakers and managers, the strategic plan faces any key changes at all, but we will see changes in sub-goals. | Given a large number of stakeholders involved in strategic plan preparation and periodic stakeholder surveys over the years of the program's implementation as well as the creation of communication channels for stakeholder feedback, it is thought that with the change of policymakers and managers, the strategic plan will remain unchanged. Keep backing the way forward. There is also a stakeholder network that, while receiving feedback from members, infuses the spirit of the strategic plan. |  | 10/12 | 0.67 | 0.92 |
| 1.3 | The Center's Planning Board is composed of members with diverse backgrounds who have specific knowledge and expertise related to the various aspects (clinical, legal, educational) related to 1,2,3,4,5,6,7 that meet quarterly at the Center's strategic meetings. They are also in constant contact with the Ministry of Health's Board of Directors and hold themselves responsible for the center 's work on 1,2,3,4,5,6,7. | The center does not have a specific planning board for 1,2,3,4,5,6,7. | The center has a planning board that does not have specialized knowledge in various areas (nutrition, management, legal affairs, health care, etc.). They are selected solely based on availability, and occasional meetings of the Planning Board are usually without specific purpose or agenda. | The Planning Board of the center selected purposefully based on appropriate knowledge and expertise in the various areas (finance, legal and tax affairs, inter-sectoral communications, etc.) that review the performance of the Center and the country each season during a meeting and direct and support the performance of the center.  In some cases, the decision-making body will consult with specialized agencies within the departments of the Ministry of Health to meet the needs. | The Planning Board selected purposefully based on the relevant knowledge and expertise in the various areas (finance, legal and tax affairs, cross-border communications, etc.) that needed to lead the strategic plan. As they review the center's executive performance each season and review its executive and financial reports, approve the center's annual work program and budget, the Planning Board also evaluates the overall performance of the Ministry of Health and themselves as a set. They are essential guides for the center.  In some cases, the decision-making body will consult with specialized agencies within the departments of the Ministry of Health to meet the needs. |  | 11/12 | 0.83 | 0.92 |

**Domain 2:** **Organizationl Management**

Questions to be considered for Organizationl Management include the following sub-domain: 2.1 The organizational structure of the NCNCD, 2.2 The organizational structure of the Ministry of Health, and 2.3 organizational procedures [36-43]. This examines the status of the appropriate organizational structure in the Ministry of Health and the NCNCD, and identifies the existence of an appropriate Organizational Structure for Service providers. The potential need to strengthen the organizational structure at NCNCD and its units is examined, as well as units that have a parallel workflow in the MoHME structure. On the other hand, the extent of NCNCD's powers in determining the appropriate structure for service provision is also measured.

**Table 7. Sub-domains of organizationl management and content validity degree**

|  |  | Low Capacity 1 | Basic Capacity 2 | Moderate Capacity 3 | Strong Capacity 4 | Score | Content Validity | | |
| --- | --- | --- | --- | --- | --- | --- | --- | --- | --- |
|  |  |  |  |  |  |  | Ne / N | CVR | I-CVI |
| 2.1 | The organizational chart that defines the reporting lines is in the NCNCD, as appropriate with the strategic plan, there are specific units associated with 1,2,3,4,5,6,7. that are reviewed annually and updated as needed. The provider structure and chart are designed for interventions related to 1,2,3,4,5,6,7.in urban and rural areas according to the strategic plan and has the potential to provide interventions in all areas (rural, suburban, nomadic, etc.). In this structure, the role of the private sector is directed and involved in providing relevant interventions. | There is no defined organizational structure; or it exists informally and there is no formal agreement on it, reporting lines, authority, and responsibility centered around the NCNCD's chair. A coherent organizational structure for urban and rural areas has not been designed, established, and formalized for interventions related to 1,2,3,4,5,6,7. | There is a written organizational structure, but the lines of reporting and oversight are not completely clear; the responsibilities and responsibilities are concentrated on a small number of characters. Organizational charts for staff at various levels are not widely known. Different structures in different provinces are providing interventions related to 1,2,3,4,5,6,7, and there is no consensus on the appropriate structure. | There is a written and formal organizational structure with clear reporting lines and oversight relationships. Organizational charts are widely known to employees at various levels. The organizational chart is aligned with the strategic plan and goals for 1,2,3,4,5,6,7. The organization chart is not updated on time. The organizational structure for delivering interventions in urban and rural areas has been identified, but there is a considerable gap in implementation. Private sector involvement and guidance have been to some extent seen in this structure. | There is a written and formal organizational structure with clear reporting lines and oversight relationships. Reporting lines and oversight relationships are defined. The organizational chart for employees at various levels is well known. The organizational chart is a reflection of the goals of the organization's strategic plan. Organization charts are regularly reviewed and updated and are constantly used. The structure of providing interventions related to 1,2,3,4,5,6,7, in urban and rural areas has been identified and implemented well. Private sector involvement and guidance in this structure have been well documented. |  | 11\12 | 0.83 | 0.83 |
| 2.2 | The organizational chart of the Ministry of Health, which contains the NCNCD, is designed so that 1,2,3,4,5,6,7-related departments are subdivided into the center and are required to follow the goals of the center based on organizational alignment lines. The main center that announces formal policies on noncommunicable diseases and their risk factors is the NCNCD. | The Ministry of Health has not released a clear organizational chart. | The NCNCD's position on the organizational chart of the Ministry of Health is very vague. Many centers decide on 1,2,3,4,5,6,7-related issues without any coordination. | In the organizational structure of the Ministry of Health, most cases of noncommunicable diseases are covered by the NCNCD. But in some cases, other centers are the main decision-makers that do not work well with the NCNCD. | All 1,2,3,4,5,6,7-related items fall under the NCNCD-related organizational structure. The NCNCD is the primary center for decision-making and communication of 1,2,3,4,5,6,7 policies. |  | 12/12 | 1.0 | 0.92 |
| 2.3 | The organizational structure of 1,2,3,4,5,6,7 related service providers is defined in urban and rural areas, where different levels of service provider population are identified and communication or referral lines are established between them. The NCNCD has been one of the key policymakers in defining this structure. | The organizational structure of the 1,2,3,4,5,6,7 service provider is unclear and the NCNCD does not have much power to determine it. | The organizational structure of the 1,2,3,4,5,6,7 service provider exists informally and the NCNCD does not have much power to determine it. | The organizational structure of the 1,2,3,4,5,6,7 service provider is formally established and it needs reform, the NCNCD has moderate authority over its structure. | The organizational structure of the 1,2,3,4,5,6,7 service provider is officially in place, and the NCNCD has a great deal of power in defining its structure.  And it does not need major reforms and meets the needs. |  | 12/12 | 1.0 | 0.83 |

**Domain 3:** **Human Resource Management**

Questions to be considered for Human Resource Management include the following sub-domain: 3.1 Human Resource Indicators, 3.2 education of Human Resource, 3.3 non-clinical professionals Human Resource [36, 44-47] Supply and training of human resources, and in particular the presence of specialized clinical human resources for 1, 2, 3 and 7 and the non-clinical specialist human resources for 4, 5, and 6 are evaluated here in this domain. Inequality in the distribution of manpower and the extent of NCNCD's authority to target these are other issues addressed here.

Table 8. Sub-domains of human resource management and content validity degree

|  |  | Low Capacity 1 | Basic Capacity 2 | Moderate Capacity 3 | Strong Capacity 4 | Score | Content Validity | | |
| --- | --- | --- | --- | --- | --- | --- | --- | --- | --- |
|  |  |  |  |  |  |  | Ne / N | CVR | I-CVI |
| 3.1 | Human Resource Indicators are defined to provide interventions related to 1,2,3,4,5,6,7 gaps identified between current and ideal status (the number of human resources required to provide current and future services). The inequality in the geographical distribution of the human resources of the service provider is calculated and commensurate with the results obtained in collaboration with the Ministry of Health. Subsequently, the number of health centers providing interventions are planned for the presence of staff at the provincial and county level. | The NCNCD does not have the authority to determine policies for providing human resources for interventions related to obesity, physical activity, and hypertension, and this is done independently by other units of the Ministry of Health. | Indicators defined, gaps determined, inequalities measured in the geographical distribution of the 1,2,3,4,5,6,7 -related workforce. They have all been informed by the decision making body at the Ministry of Health, but the actions and decisions of the decision-making body are not based on the needs and these facts. The number of centers required to provide interventions at the provincial and county levels is not planned. | Indicators are defined, gaps are defined, inequalities are measured in the geographical distribution of the 1,2,3,4,5,6,7 -related workforce. All of them have been informed by the decision-making body at the Ministry of Health. The Ministry of Health's decision-making and human resources decision-making body applies some of the information reported in their decisions. The number of centers required to provide interventions at the provincial and county level is planned. | Indicators defined, gaps defined, inequalities measured in the geographical distribution of the 1,2,3,4,5,6,7 -related workforce. All of them have been informed by the decision-making body at the Ministry of Health. The decision-making body of the Ministry of Health largely implements its decisions. The number of centers required to provide interventions at the provincial and county level is planned and is being implemented. |  | 12/12 | 1.0 | 0.83 |
| 3.2 | The development of knowledge and education of service providers (professors, students, and service providers) in 1,2,3,4,5,6,7 -related fields is generally ongoing across the country and educational resources are being updated annually. | The NCNCD does not work on human resources training policies or the NCNCD does not have the authority to determine 1,2,3,4,5,6,7 training policies. | The NCNCD has prepared human resources training headlines and policies, but the Department of Education (M0HME) has not put them on the agenda and is reviewing them with a long break. | The NCNCD prepares human resources training headings and policies, and the Department of Education (MOH) partially puts them on the agenda and reviews them at an acceptable interval. But the results of the change in training are not clear. | The NCNCD provides human resources training headings and policies and the Department of Education (M0HME) puts them all on the agenda and reviews them immediately. The effects of change in training are clear and visible. |  | 11/12 | 0.83 | 0.92 |
| 3.3 | Non-clinical/clinical professionals with sufficient knowledge of,4,5,6 / 1,2,3,7 to develop non-clinical / clinical interventions, including mass media, group training, interdisciplinary interventions, and legislation/ clinical are available to the NCNCD. and the NCNCD Planning Board is aware of the importance of this kind of force. | The NCNCD does not have written, formal or oral or informal policies for the development and use of non-clinical specialized human resources in interventions related to 1,2,3,4,5,6,7. | The NCNCD has written, formal or oral and informal policies on the development and use of non-clinical specialized human resources in interventions related to 1,2,3,4,5,6,7. But they are not usually followed up and/or the NCNCD has usually experienced the use of non-specialized forces in these areas. The Center does not have the authority to employ the required non-clinical specialized personnel. | The NCNCD has specific written and formal policies on the development and use of non-clinical specialized force in interventions related to 1,2,3,4,5,6,7, and most of these policies are being followed nationally. And the Center has the authority to deploy specialized non-clinical personnel. | The NCNCD has specific written and formal policies on the development and use of non-clinical specialized force in interventions related to 1,2,3,4,5,6,7,, and most of these policies are being followed nationally. These policies have been pursued and specialized human resources are operating in these areas at the national and provincial levels. And the Center has the authority to deploy specialized non-clinical personnel. |  | 10/12 | 0.67 | 0.75 |

**Domain 4:** **Financial Management**

The financial management dimension [2, 36, 48-55] measures the status of finance interventions and protects people from high out-of-pocket *costs* for a *catastrophic health* event, how to allocate resources, and how to pay providers. The extent of NCNCD's powers in the area of financial management of noncommunicable disease management is obtained through this dimension.

**Table 9. Sub-domains of Financial Management and content validity degree**

|  |  | Low Capacity 1 | Basic Capacity 2 | Moderate Capacity 3 | Strong Capacity 4 | Score | Content Validity | | |
| --- | --- | --- | --- | --- | --- | --- | --- | --- | --- |
|  |  |  |  |  |  |  | Ne / N | CVR | I-CVI |
| 4.1 | The Center has written policies and procedures for financing obesity-related interventions. The flow of financial resources and the indicators of distribution of resources to each domain are identified. Various funding sources (insurance, public and private taxes, public funds, NGOs, etc.) have been identified to financing the implementation of related interventions. | The Center has no funding policies or procedures in this area, or the Center has informal policies and procedures that are not in written format, or most financial policies and procedures are outdated. Sources of funding for interventions have not been identified. Or the center has little authority in determining financing policies. | The NCNCD has outlined 1,2,3,4,5,6,7 financing policies and procedures, but they are not completely clear. Often financial performance is not in line with policies and procedures. The sources of funding for the interventions are not clearly defined and detailed, and some of them are outdated. Center for Preventive Intervention Financial Services has a maximum of consultative status. | The NCNCD has written 1,2,3,4,5,6,7-related financial policies and procedures in a handbook that is easily accessible to staff and providers.  Policies and procedures are following generally accepted accounting principles (GAAP).  Financial performance is usually in line with established policies and procedures. The sources of funding for interventions are mostly clear. | The organization has a finance guide that addresses obesity, physical activity, is readily available and directs staff.  Policies and procedures are fully compliant with GAAP. The funding sources are quite clear.  Financial performance is always in line with financial policies and procedures. The sources of funding for interventions are quite clear. |  | 12/12 | 1.0 | 1.0 |
| 4.2 | The allocation of funds to interventions related to 1,2,3,4,5,6,7 against other domains has included economic and social criteria such as cost effectiveness, promotion of health equity, insurance policies (being covered). Financial protection of community members against 1,2,3,4,5,6,7 related health services has been seen in the center's financing policies. | The allocation of resources is not within the jurisdiction of the Center and NCNCD has not yet established a policy in this regard.  Prioritizing resources is not done. There has been no discussion of protecting the community from the health costs associated with 1,2,3,4,5,6,7 -dservices. | Resource allocation policies have been formulated orally and have been communicated to higher authorities. There has been no discussion of the financial protection from the health costs associated with 1,2,3,4,5,6,7, services but is under investigation. | Policies for resource allocation to interventions have been formulated according to socioeconomic and health criteria, but allocation practices are usually different from those policies. Financial protection has been provided to people in the community about 1,2,3,4,5,6,7 services. And in practice, vulnerable people have been well protected from the health costs associated with 1,2,3,4,5,6,7, services | Intervention resource allocation policies are defined and written according to socioeconomic and health criteria, and based on the criteria considered, resource allocation priorities are identified and allocation performance complies with these policies. Financial protection has been provided to people in the community about obesity, physical activity and blood pressure services. And in practice, people are best protected from associated health costs. |  | 12/12 | 1.0 | 0.92 |
| 4.3 | Optimal methods of payment to providers of interventions (clinical, educational, counseling, etc.) related to 1,2,3,4,5,6,7 have been established. Appropriate reimbursement methods for expanding the provision of such services have been identified and applied. | It is not within the competence of the Center to determine payment methods to providers and so far has not conducted any studies on this. | Payment policies for interventions providers are verbally defined and at the center of attention and have not yet been written, and sporadic studies have been conducted. | Providers' payment policies and methods have been reviewed and formulated, formally written, and guide the organization's plans. The payment practices are also partly in line with these policies and procedures. | . Providers' payment policies and procedures have been reviewed and legally written. And it is a guide to planning and payments. Payment practices are fully in line with established policies and procedures. |  | 11/12 | 0.83 | 0.92 |

**Domain 5:** **Program / Project Management**

Dimension 5 measures the relevance of programs and projects to the strategic plan[36, 56, 57], also, the status of evaluations of programs and projects[1, 58-60], and Linking programs with other major programs, including PHC and family physician [61, 62], surveillance systems and standards of service delivery [63-65], and their prioritization are among the most important questions in this dimension.

**Table 10. Sub-domains of program / project management and content validity degree**

|  |  | Low Capacity 1 | Basic Capacity 2 | Moderate Capacity 3 | Strong Capacity 4 | Score | Content Validity | | |
| --- | --- | --- | --- | --- | --- | --- | --- | --- | --- |
|  |  |  |  |  |  |  | Ne / N | CVR | I-CVI |
| 5.1 | Does the Center conduct or direct 1,2,3,4,5,6,7-related programs in line with the country's strategic plan, and conduct periodic review sessions to report results, share knowledge and experiences, and, where appropriate, review the strategy.  Program evaluation is done through methods such as cost-effectiveness, health impact assessment, gender, and income impact assessment, multi-criteria analysis, etc. through self-assessment and other evaluation. | 1,2,3,4,5,6,7-related programs are not implemented in line with the strategic plan or there is no relationship between the implementation of the programs and the achievement of the strategic plan goals and there are no review meetings in this regard. Monitoring how programs are executed is done informally or review meetings are rarely held and programs are not usually evaluated. | The center typically runs 1,2,3,4,5,6,7-related programs in line with strategic plans, but sometimes deviates from them. Review sessions are held in specific sessions, attended by service providers, where knowledge, learning, and experiences are discussed and exchanged. Evaluation of programs based on personal opinions and lack of evaluation by outside people (Other evaluation) and less tool based. | 1,2,3,4,5,6,7 programs are implemented to achieve the goals of the strategic plan, and regular review sessions are held with staff and providers and their feedback. In that knowledge, learning, and experiences are discussed and exchanged. Program evaluation is based on methods such as cost-effectiveness, health impact assessment, gender and income impact assessment, multi-criteria analysis, etc., and usually, follow principles that include self- evaluation and other evaluation. | The NCNCD has a strategic plan that is used to guide the implementation of the program and the programs are implemented to achieve it.  Program review sessions are often attended by staff, service providers, service recipients (or their feedback) and other stakeholders, while knowledge, experience, and lessons learned are discussed and strategic planning is provided as needed. Program evaluation programs are based on methods such as cost-effectiveness, health impact assessment, gender, and income impact assessment, multi-criteria analysis, etc. and are always followed, including self-assessment and other will be evaluated. |  | 10/12 | 0.67 | 0.83 |
| 5.2 | The 1,2,3,4,5,6,7 Information System measures 1,2,3,4,5,6,7 -related incidence and prevalence rates at the national, provincial, and local levels; (Income levels) combined. The amount of services provided and their outcomes (indicators such as preserved life, increased labor productivity in the country, increased life expectancy, economic savings) are reported to national policymakers. The information needs of the program have been identified and communicated to the Ministry of Health's Information and Technology Management Center. | The Center has not designed its own dedicated information system in this area. | The center has an outdated information system that gathers information on prevalence and incidence in the area based on a variety of practices and resources in each region. The information system is not connected to higher information systems such as your GP and referral system. The information needs of the center for better program management have not been identified. | The Information System Center has recently reviewed that prevalence and incidence information is obtained in areas based on the same guidelines. The information system is linked to higher information systems such as the GP and referral system, the information needs of the center have been identified for better program management, and have been communicated to the Ministry of Health's Information and Technology Management Center. The impacts and results of the programs are reported to national policymakers in the form of economic savings indicators, increased life expectancy, increased labor productivity, etc. | The center has an updated information system that is reviewed annually as needed. Based on that, prevalence and incidence data in the regions are obtained according to the same guidelines. Information system is connected to higher information systems such as physician and referral system, family decks. Information center needs for better program management have been identified and submitted to Ministry of Health and Higher Information Council Information and Technology Management Center and needs are appropriately addressed. have became. The impacts and results of programs are reported to national policymakers in the form of economic savings indicators, increased life expectancy, increased labor productivity, and so on. |  | 12/12 | 1.0 | 0.92 |
| 5.3 | The Center has developed quantitative and qualitative standards for 1,2,3,4,5,6,7 -based interventions that are based on international recommendations and successful worldwide experiences and are monitored. Required interventions, medications and diagnostic equipment are identified and are being provided, and community, users and providers are satisfied with the status of services provided. | There are no qualitative and quantitative standards for nutrition-related interventions.  How the service is provided is not monitored. Qualitative and quantitative standards exist informally or are outdated. Interventions, medicines and diagnostic and therapeutic equipment needed are not specified. We usually face shortages. Service providers and recipients are often sorrowful. | There are written and quantitative standards for some of the interventions being offered. But not for all of them. They are not quite clear. The interventions, drugs, and diagnostic and therapeutic equipment needed are unclear and exact statistics are not provided.  It is believed that the center is making positive changes but cannot produce definite results. Recipients of the service are somewhat satisfied. | Written quantitative and qualitative standards have been developed for many nutrition-related interventions and services. These standards address the needs of the community/clients and are generally in line with global standards. Most of the interventions, drugs, and diagnostic and therapeutic equipment needed to provide services have been identified and are partially being funded.  The center monitors standards.  Service recipients have expressed satisfaction with the services provided. | Written quantitative and qualitative standards have been developed for all interventions and services being provided. These standards address the needs of the community/clients and comply with global standards. All required interventions, medications and diagnostic equipment are identified and prioritized and are being funded.  The Center continuously monitors compliance with standards.  The recipients of services have mostly expressed satisfaction with the services provided. |  | 11/12 | 0.83 | 0.83 |

Dimension 6: External and Internal relations

In this section, we analyze the capacity of the Center for inter-sectoral and intera-sectoral Relations as well as how policies can be transformed into laws. [36, 66-73]

**Table 11. Sub-domains of external and internal relations and content validity degree**

|  |  | Low Capacity 1 | Basic Capacity 2 | Moderate Capacity 3 | Strong Capacity 4 | Score | Content Validity | | |
| --- | --- | --- | --- | --- | --- | --- | --- | --- | --- |
|  |  |  |  |  |  |  | Ne / N | CVR | I-CVI |
| 6.1 | The center has an external relations strategy (with individuals or organizations outside the Ministry of Health) to develop 1,2,3,4,5,6,7 -related interventions and uses it to communicate effectively with key stakeholders. The relevant inter-departmental councils (Supreme Council for Statistics, Insurance, Health, Standards, etc.) have been identified and contacted through a representative of the Ministry of Health. | The center itself does not have cross-sectional relations or the external relations of the center are informally defined and there is no formal definition of them. | The center has a simple external communication strategy that is formalized/documented, but not comprehensive or widely recognized. Some attempts to identify and communicate with key stakeholders have been successful, but most have not succeeded. The relevant cross-border councils have not been identified. | The center has an external communication strategy that is formal / documented and comprehensive, recognized by most staff. Key points, areas, and ways to connect with stakeholders have been identified and are ongoing. The Center conducts informal stakeholder analysis and identifies stakeholders at national level. Related transboundary councils have been identified but have not been contacted. | The center has an external relations strategy that is formalized/documented, comprehensive, widely recognized by all staff, and regularly reviewed. Key areas for communication with different stakeholder groups have been identified and are in constant contact with them. The Stakeholder Analysis Center is specialized and identifies stakeholders at the provincial and national levels; stakeholder analysis is regularly updated. Related transboundary councils have been identified. |  | 12//12 | 1.0 | 1.0 |
| 6.2 | The center has an internal relations strategy (with individuals or departments and organizations under the Ministry of Health) to develop 1,2,3,4,5,6,7 -related interventions and uses it to communicate effectively with key stakeholders. The stakeholder information network is set up inside and outside the organization and the key messages are communicated to the stakeholders and feedback is received. | The center has no internal relationships. Or those internal relationships are defined informally and there is no formal definition on them. | The center has a simple intra-organizational communication strategy that is formal / documented, but not comprehensive or widely recognized.  Some efforts have been made to identify and communicate with key stakeholders within the organization, but most have not been successful. | The organization has an in-company communication strategy that is formal / documented and comprehensive, recognized by most employees. Key points, areas, and ways to connect with other departments within the Ministry of Health have been identified and are ongoing. NCNCD has performed an initial stakeholder analysis within the organization and identified stakeholders at the national level. A stakeholder communication network is established but not very active. | The organization has an organization-wide relationship strategy that is formalized / documented, comprehensive, widely recognized by all staff, and regularly reviewed.  Key areas for communication with the various departments of the Ministry of Health have been identified and are in constant contact with them. The Center conducts stakeholder analysis and identifies stakeholders at the provincial and national levels; stakeholder analysis is regularly updated. Inter-organizational coordination councils have been identified. A network of inter-organizational and external stakeholder networks has been established and is very active and productive. |  | 12/12 | 1.0 | 0.92 |
| 6.3 | The organization has identified the process and paths for converting its proposed policies into laws in cases where it needs to make its proposed policies in the field of 1,2,3,4,5,6,7 to a general law. | The center does not know a clear path in the process of converting policies into laws. Or, it does not consider the task of the Center to make the proposed policies into laws. | The Center uses informal paths (informal communication with empowered persons) in the process of converting policies to laws. These paths are based on personal opinion. | The Center has identified and documented formal pathways for the conversion of 1,2,3,4,5,6,7 policies into laws. These routes are known by most staff and are often used. | The Center has identified and documented formal pathways for the conversion of 1,2,3,4,5,6,7 policies into laws. These routes are known by all staff and are always used and are reasonable and productive routes. |  | 10/12 | 0.67 | 1.0 |

- **Result for each subjects (main domain and sub-domain)**

**1. Cardiovascular disease and hypertension**


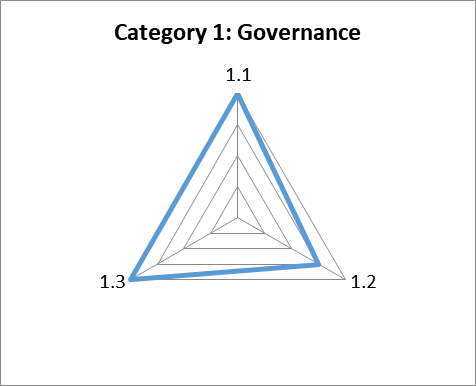

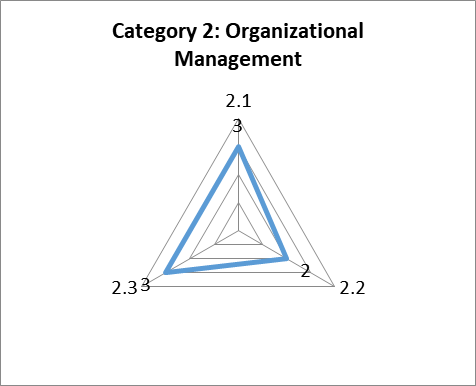

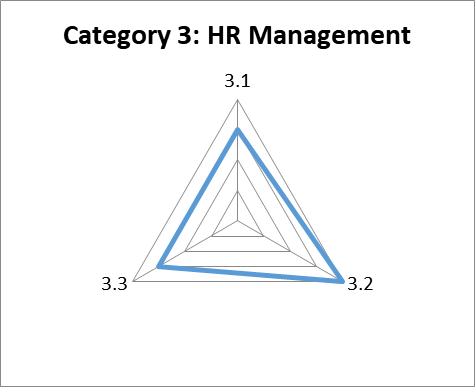

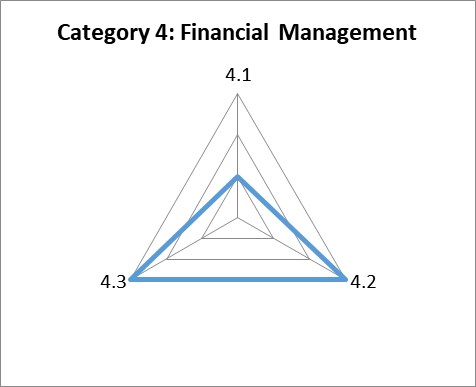

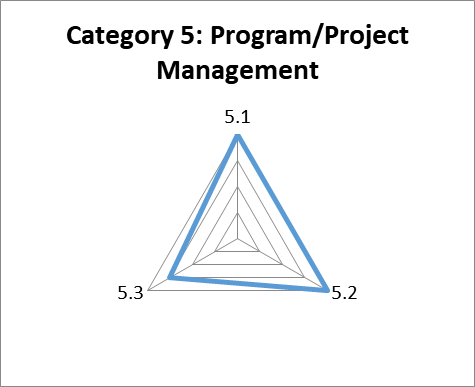

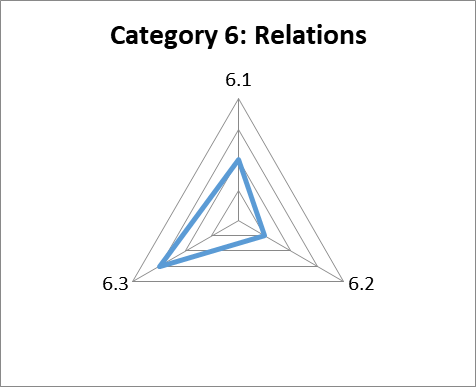


**F****ig 3. The capacity assessment results, Cardiovascular disease and hypertension**

**2.** **Diabetes**


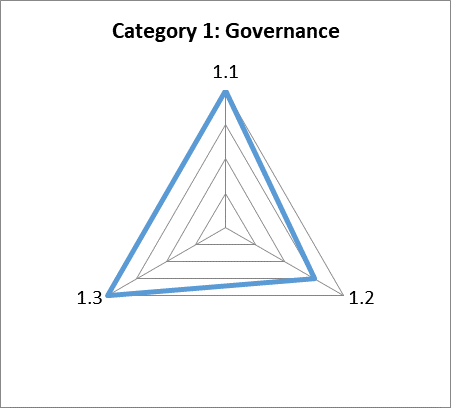

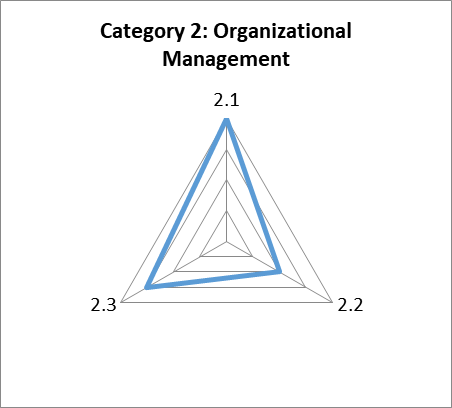

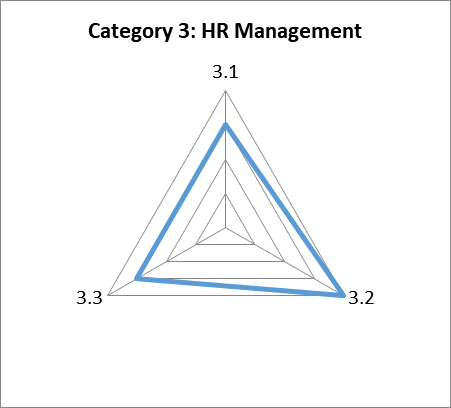

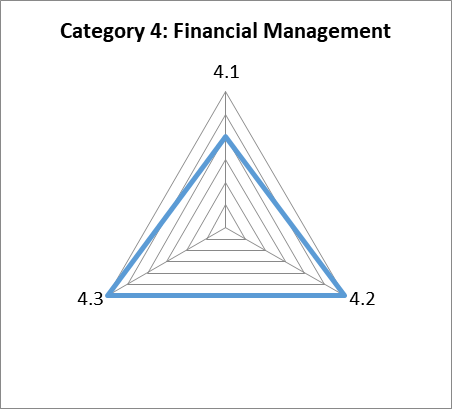

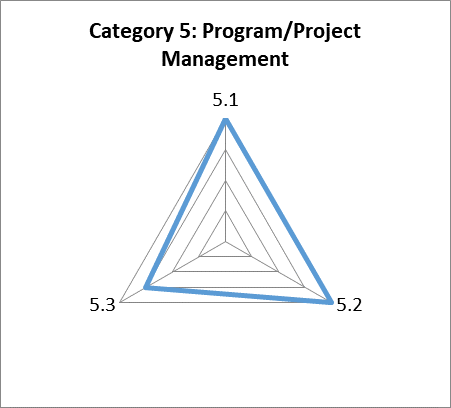

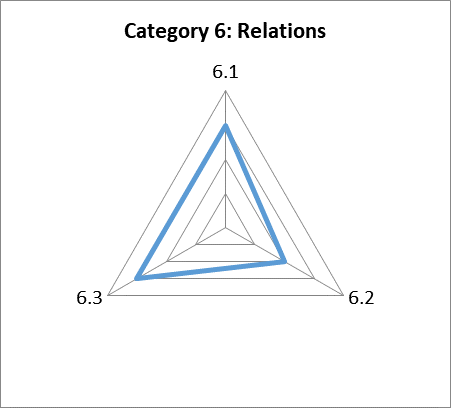


**Fig 4. The capacity assessment results, Diabetes**

**3.** **Chronic Respiratory Disease**


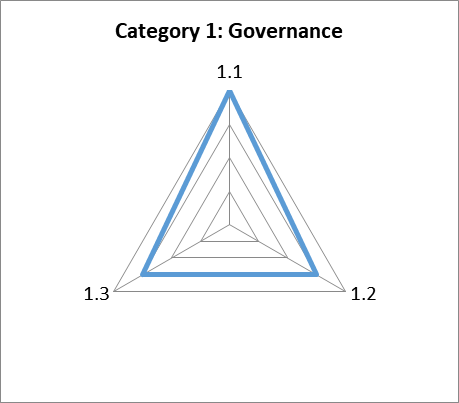

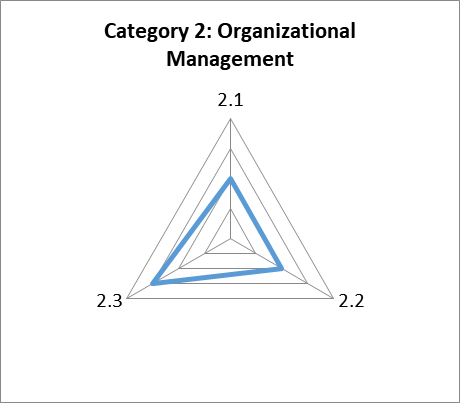

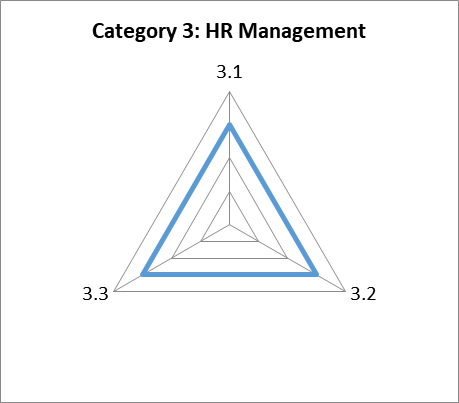

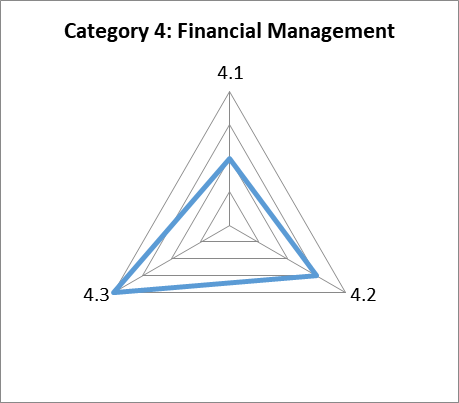

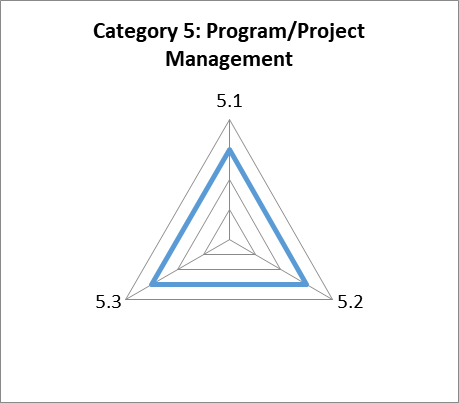

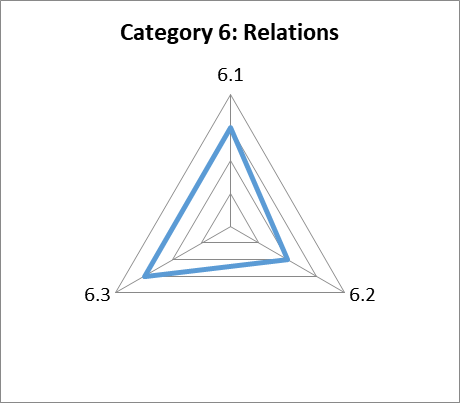


**Fig 5. The capacity assessment results, Chronic Respiratory Disease**

**4.** **Obesity and Physical Activity**


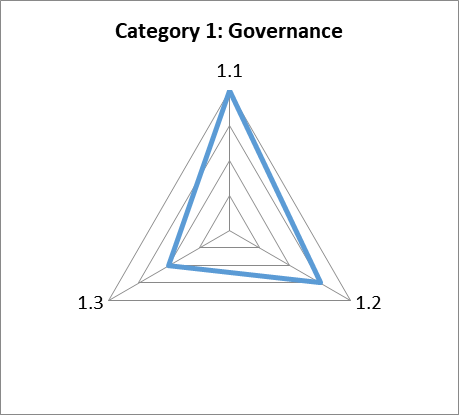

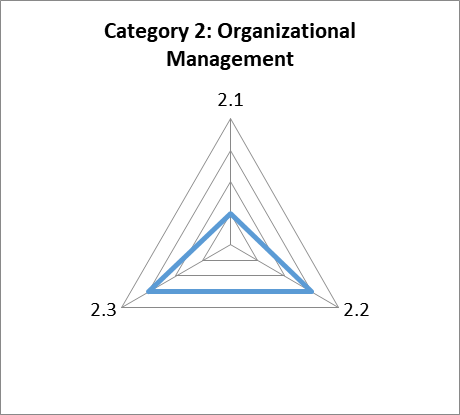

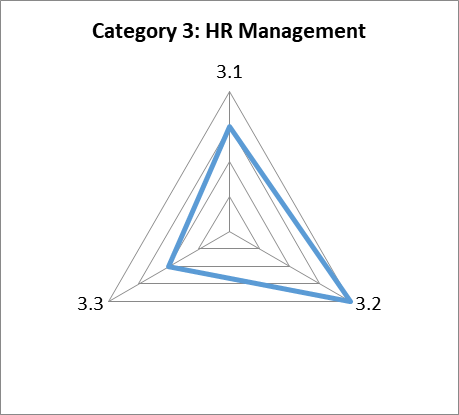

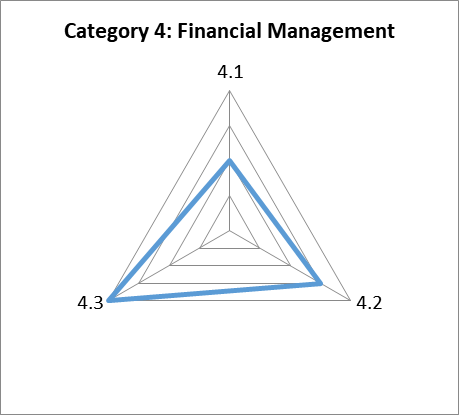

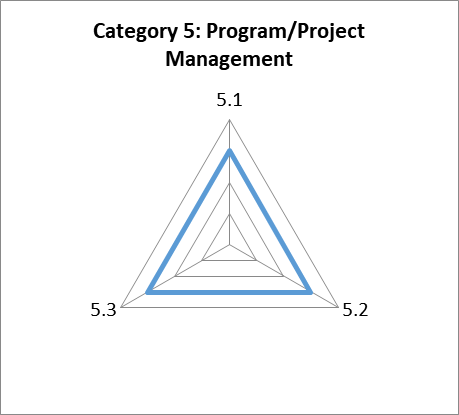

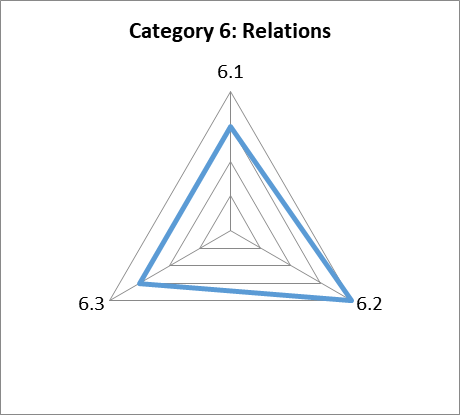


**Fig 6. The capacity assessment results,** **Obesity and Physical Activity**

**5.** **Tobacco and Alcohol**


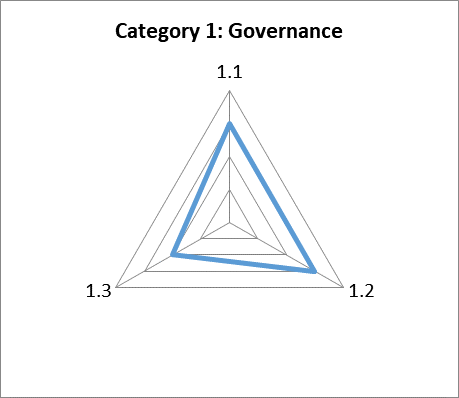

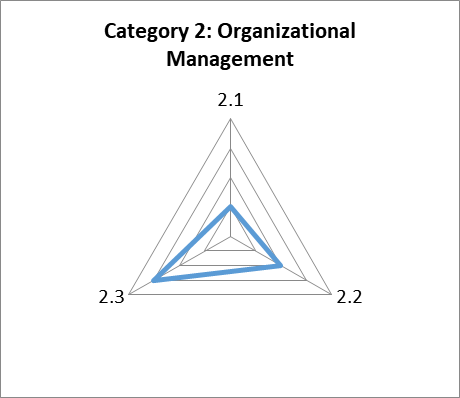

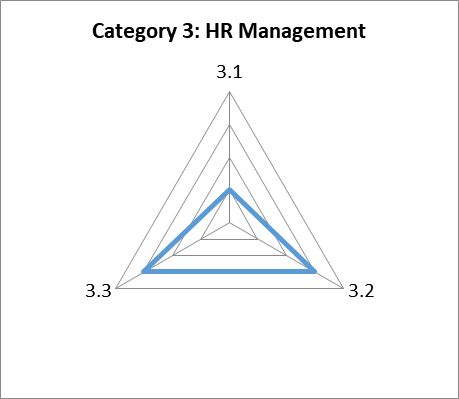

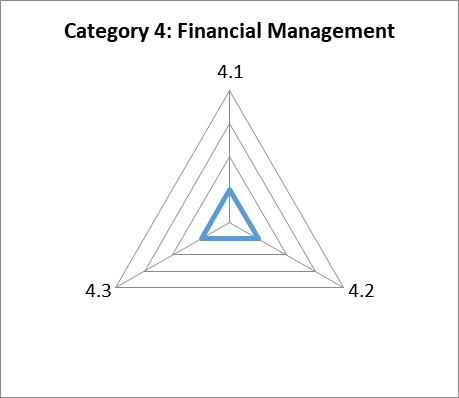

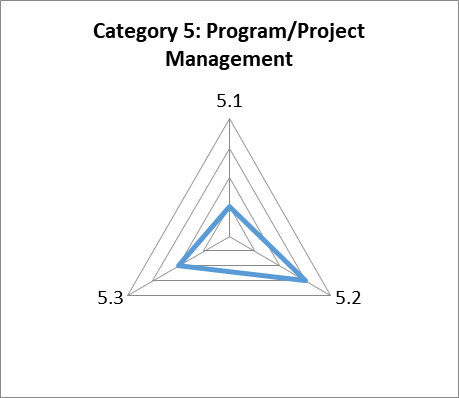

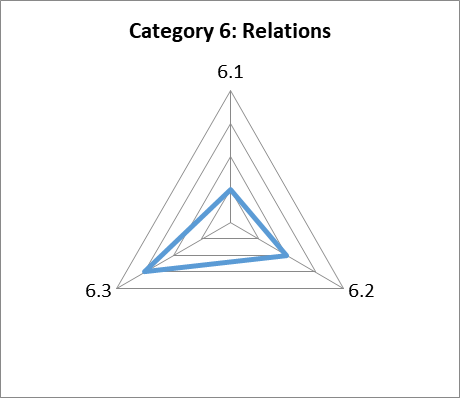


**Fig 7. The capacity assessment results,** **Tobacco and Alcohol**

**6. Nutrition (salt, sugar, fat, fruit and vegetable)**

**Fig 8. The capacity assessment results, Nutrition**

**7. Cancer**

**Fig 9. The capacity assessment results, cancer**

**References:**

1. Organization WH. Assessing national capacity for the prevention and control of noncommunicable diseases: report of the 2015 global survey. 2016.

2. Shams L, Yazdani S, Takian A, Nasiri T. Multi-sectoral Requirements of Non-Communicable Diseases Stewardship in Iran. J Revista Publicando. 2018;5(15):1420-37.

3. Organization WH. Implementation tools: package of essential noncommunicable (‎ PEN)‎ disease interventions for primary health care in low-resource settings: World Health Organization; 2013.

4. WHO. Accelerating regional implementation of the Political Declaration of the Third High-level Meeting of the General Assembly on the Prevention and Control of Noncommunicable Diseases, 2018. 2019.

5. Organization WH. Service availability and readiness assessment (SARA): an annual monitoring system for service delivery: reference manual. World Health Organization; 2013.

6. Vaidya A. Capacity building: A missing piece in Nepal’s plan for prevention and control of non-communicable diseases. Journal of Kathmandu Medical College. 2018 Dec 31;7(4):131-3.

7. OECD. Implementation guidelines on evaluation and capacity building for the local and micro regional level 2009.

8. Austrian Development Agency, Evaluation Unit; Guidelines for Project and Programme Evaluations. 2009.

9. Pact, Organizational Capacity Assessment (OCA) Handbook; A practical guide to the oca tool for practitioners and development professionals. 2012. <https://www.pactworld.org/sites/default/files/OCA%20Handbook_ext.pdf>.

10. Bateson DS, Lalonde AB, Perron L, Senikas V. Methodology for assessment and development of organization capacity. J Obstet Gynaecol Can. 2008;30(10):888-95.

11. Westfall CT. Got Inclusion? How Inclusive is your Organization? Assessing Inclusion through the iCAT: Inclusion Capacity Assessment Tool for Organizational Capacity. 2016.

12. Snow J. Organizational Capacity Assessment Tool - John Snow, Inc. 2014.

13. USAID. The USAID Organizational Capacity Assessment (OCA) Tool 2016 [Available from: <https://usaidlearninglab.org/library/organizational-capacity-assessment>.

14. Assessing national capacity for the prevention and control of noncommunicable diseases: report of the 2017 global survey. Geneva: World Health Organization; 2018. Licence: CC BYNC-SA 3.0 IGO.

15. Bourgeois I, Cousins JB. Understanding dimensions of organizational evaluation capacity. J American Journal of Evaluation. 2013;34(3):299-319.

16. Informing-Change. A Guide to Organizational Capacity Assessment Tools. 2017.

17. Zamanzadeh V, Ghahramanian A, Rassouli M, Abbaszadeh A, Alavi- H. Design and implementation content validity Study : development of an instrument for measuring patient-centered communication. J Caring Sci. 2015;4(5):165–78.

18. Lawshe CHJPp. A quantitative approach to content validity 1. 1975;28(4):563-75.

19. DuBois R, Bruce K, Reeves M, Vandelanotte J, Yakimakho OJPIQ. The Organizational Performance Index: A New Method for Measuring International Civil Society Capacity Development Outcomes. 2019;31(4):381-96.

20. Team RC. R: A Language and Environment for Statistical Computing. 2019.

21. Signorell A, Aho K, Alfons A, Anderegg N, Aragon T, Arppe A, et al. DescTools: Tools for descriptive statistics. R package version 0.99. 28. 2019.

22. Meyer AM, Davis M, Mays GP. Defining organizational capacity for public health services and systems research. J Public Health Manag Pract. 2012;18(6):535-44.

23. Barbazza E, Tello JEJHp. A review of health governance: definitions, dimensions and tools to govern. 2014;116(1):1-11.

24. Kaufmann D, Recanatini F, Biletsky SJWBIDD, Washington, DC. Assessing governance: diagnostic tools and applied methods for capacity building and action learning. 2002.

25. Siddiqi S, Masud TI, Nishtar S, Peters DH, Sabri B, Bile KM, et al. Framework for assessing governance of the health system in developing countries: gateway to good governance. 2009;90(1):13-25.

26. Kanie N, Biermann F. Governing through goals: Sustainable development goals as governance innovation: mit Press; 2017.

27. Van de Pas R, Hill PS, Hammonds R, Ooms G, Forman L, Waris A, et al. Global health governance in the sustainable development goals: Is it grounded in the right to health? 2017;1(1):47-60.

28. Kaufmann D, Kraay A. Governance indicators: Where are we, where should we be going?: The World Bank; 2007.

29. Savedoff WD. Governance in the health sector: a strategy for measuring determinants and performance: The World Bank; 2011.

30. Moldan B, Billharz S, Matravers R. Sustainability indicators: A report on the project on indicators of sustainable development: Wiley New York; 1997.

31. Malekafzali HJIJPH. Primary health care in the rural area of the Islamic Republic of Iran. 2009;38(Suppl 1):69-70.

32. Lewis M, Pettersson G. Governance in health care delivery: raising performance: The World Bank; 2009.

33. Smith PC, Anell A, Busse R, Crivelli L, Healy J, Lindahl AK, et al. Leadership and governance in seven developed health systems. 2012;106(1):37-49.

34. Veillard JHM, Brown AD, Barış E, Permanand G, Klazinga NSJHP. Health system stewardship of National Health Ministries in the WHO European region: concepts, functions and assessment framework. 2011;103(2-3):191-9.

35. Saltman RB, Duran AJIjohp, management. Governance, government, and the search for new provider models. 2016;5(1):33.

36. Bonita R, Magnusson R, Bovet P, Zhao D, Malta DC, Geneau R, et al. Country actions to meet UN commitments on non-communicable diseases: a stepwise approach. 2013;381(9866):575-84.

37. Beitsch LM, Brooks RG, Grigg M, Menachemi NJAJoPH. Structure and functions of state public health agencies. 2006;96(1):167-72.

38. Hyde JK, Shortell SMJAjopm. The structure and organization of local and state public health agencies in the US: a systematic review. 2012;42(5):S29-S41.

39. Unger J-P, Macq J, Bredo F, Boelaert MJBotWHO. Through Mintzberg’s glasses: a fresh look at the organization of ministries of health. 2000;78:1005-14.

40. Jeppsson A, Östergren P-O, Hagström BJHP, Planning. Restructuring a ministry of health–an issue of structure and process: A case study from Uganda. 2003;18(1):68-73.

41. Hogg W, Rowan M, Russell G, Geneau R, Muldoon LJIJfQiHC. Framework for primary care organizations: the importance of a structural domain. 2007;20(5):308-13.

42. Hakobyan T, Nazaretyan M, Makarova T, Aristakesyan M, Margaryants H, Nolte E. Armenia: Health system review. 2006.

43. Peykari N, Hashemi H, Dinarvand R, Haji-Aghajani M, Malekzadeh R, Sadrolsadat A, et al. National action plan for non-communicable diseases prevention and control in Iran; a response to emerging epidemic. 2017;16(1):3.

44. Mendis S, Al Bashir I, Dissanayake L, Varghese C, Fadhil I, Marhe E, et al. Gaps in capacity in primary care in low-resource settings for implementation of essential noncommunicable disease interventions. 2012;2012.

45. Islam SMSJIJoPiPH. Human resources for non-communicable diseases in Bangladesh. 2017;1(2):98-101.

46. Engelgau M, Okamoto K, Navaratne KV, Gopalan S. Prevention and control of selected chronic NCDs in Sri Lanka: policy options and action. 2010.

47. Nyoni J, Gbary A. Policies and plans for human resources for health: guidelines for countries in the WHO African region: WHO Regional Office for Africa; 2008.

48. Evans DB, Etienne C. Health systems financing and the path to universal coverage. SciELO Public Health; 2010.

49. Kulesher R, Forrestal EJJoHA. International models of health systems financing. 2014;3(4):127-39.

50. Ranson K, Law TJ, Bennett SJSs, medicine. Establishing health systems financing research priorities in developing countries using a participatory methodology. 2010;70(12):1933-42.

51. Kutzin JJBotWHO. Health financing for universal coverage and health system performance: concepts and implications for policy. 2013;91:602-11.

52. Fryatt R, Mills A, Nordstrom AJTL. Financing of health systems to achieve the health Millennium Development Goals in low-income countries. 2010;375(9712):419-26.

53. Thomson S, Foubister T, Mossialos E. Financing health care in the European Union: challenges and policy responses: World Health Organization. Regional Office for Europe; 2009.

54. Jakovljevic M, Jakab M, Gerdtham U, McDaid D, Ogura S, Varavikova E, et al. Comparative financing analysis and political economy of noncommunicable diseases. 2019;22(8):722-7.

55. Abolhallaje M, Ramezanian M, Abolhasani N, Salarian Zade H, Hamidi H, Bastani PJWASJ. Iranian health financing system: challenges and opportunities. 2013;22(5):662-6.

56. Biørn K, Saeed MJNUoS, Technology. The link between organizational strategy and projects. 2014.

57. Tharp J, editor Align project management with organizational strategy. PMI Global Congress Proceedings, Hong Kong; 2007.

58. Grembowski D. The practice of health program evaluation: Sage Publications; 2015.

59. Atun R, Jaffar S, Nishtar S, Knaul FM, Barreto ML, Nyirenda M, et al. Improving responsiveness of health systems to non-communicable diseases. 2013;381(9867):690-7.

60. Hogerzeil HV, Liberman J, Wirtz VJ, Kishore SP, Selvaraj S, Kiddell-Monroe R, et al. Promotion of access to essential medicines for non-communicable diseases: practical implications of the UN political declaration. 2013;381(9867):680-9.

61. Jakab M, Hawkins L, Loring B, Tello J, Ergüder T, Kontas M. Better non-communicable disease outcomes: challenges and opportunities for health systems, No. 2, Turkey Country Assessment. WHO Regional Office for Europe. 2014.

62. Varghese C, Nongkynrih B, Onakpoya I, McCall M, Barkley S, Collins TEJB. Better health and wellbeing for billion more people: integrating non-communicable diseases in primary care. 2019;364:l327.

63. Dhungana SP, Karmacharya RM, Pyakurel P, Shrestha A, Vaidya AJNHJ. Health information system as an integral component of cardiovascular surveillance system in Nepal. 2019;16(1):7-10.

64. Kassa M, Grace J. The Global Burden and Perspectives on Non-communicable Diseases (NCDs) and the Prevention, Data Availability and Systems Approach of NCDs in Low-resource Countries. Non-communicable Diseases and Urbanization-A Global Perspective: IntechOpen; 2019.

65. Organization WH. Regional consultation and partner's forum on NCD surveillance and monitoring, Bangkok, Thailand, September 17-19, 2018. World Health Organization. Regional Office for South-East Asia; 2019.

66. Organization WH. Global status report on noncommunicable diseases 2014. World Health Organization; 2014.

67. Hospedales CJ, Jané-Llopis EJJohc. A multistakeholder platform to promote health and prevent noncommunicable diseases in the region of the Americas: the Pan American Health Organization partners forum for action. 2011;16(sup2):191-200.

68. Zafar M, Malik MAJJoID, Therapy. Emerging challenges and health system capacity: the case of non-communicable diseases in Pakistan; a review. 2014;2(1):2332.

69. Magnusson R, Patterson DJL. The Role of Law in the Global Response to NCDs. 2011;378(9794):859-60.

70. McKee M, Haines A, Ebrahim S, Lamptey P, Barreto ML, Matheson D, et al. Towards a comprehensive global approach to prevention and control of NCDs. 2014;10(1):74.

71. Magnusson RSJBgh. Framework legislation for non-communicable diseases: and for the Sustainable Development Goals? 2017;2(3):e000385.

72. Reeve B, Gostin LO. “Big” Food, Tobacco, and Alcohol: Reducing Industry Influence on Noncommunicable Disease Prevention Laws and Policies. 2019.

73. United Nations GA. Political declaration of the High‐level Meeting of the General Assembly on the Prevention and Control of Non‐communicable Diseases, A/66/L. 1. 2011.
